# Histoplasmosis Causing Overt Inflammation Presenting as Pericarditis: A Case Report

**DOI:** 10.7759/cureus.59512

**Published:** 2024-05-02

**Authors:** Amanda C Yeates, David S Quimby

**Affiliations:** 1 Infectious Diseases, Creighton University, Omaha, USA; 2 Medicine/Infectious Diseases, Creighton University, Omaha, USA

**Keywords:** fungal infection, inflammatory syndrome, inflammation, pericarditis, histoplasmosis

## Abstract

*Histoplasma capsulatum* is a well-known cause of pulmonary infections, but in the right patient population or host environment, it can cause a vast array of symptoms. The fungus possesses a special set of virulence factors that allows it to evade host immunity and cause infection, particularly in immunosuppressed hosts. Pericarditis is a known presentation of histoplasmosis, but it can be difficult to diagnose and is often treated based on suspicion. We present a case of a healthy young male who mounted a robust inflammatory response to histoplasmosis resulting in pericarditis.

## Introduction

*Histoplasma capsulatum* is one of several fungi endemic to certain parts of the world that is known to cause infection in humans [1]. While histoplasmosis can remain subclinical in many, it can cause a much more aggressive infectious course in the right host. While pulmonary infections, often with generalized symptoms, are the most common clinical presentation, infection may also be disseminated or with central nervous system involvement [1]. At times, generalized symptoms may be indicative of a more widespread infection. Here, we present a case of robust inflammation caused by *Histoplasma capsulatum *manifesting as pericarditis in a previously healthy 22-year-old male.

## Case presentation

A 22-year-old man with no chronic medical conditions and taking no chronic medications developed upper abdominal and chest discomfort with breathing. He was evaluated in the emergency department where basic laboratory studies and EKG were unremarkable. He was discharged from the emergency department with recommendations to start a proton pump inhibitor for possible gastroesophageal reflux. On a follow-up visit about one week later, there was some improvement in the chest discomfort, although it had not completely resolved and was still present with deep breathing. He had a new complaint of back discomfort that was attributed to strenuous physical activity for which he was started on meloxicam.

**Figure 1:**
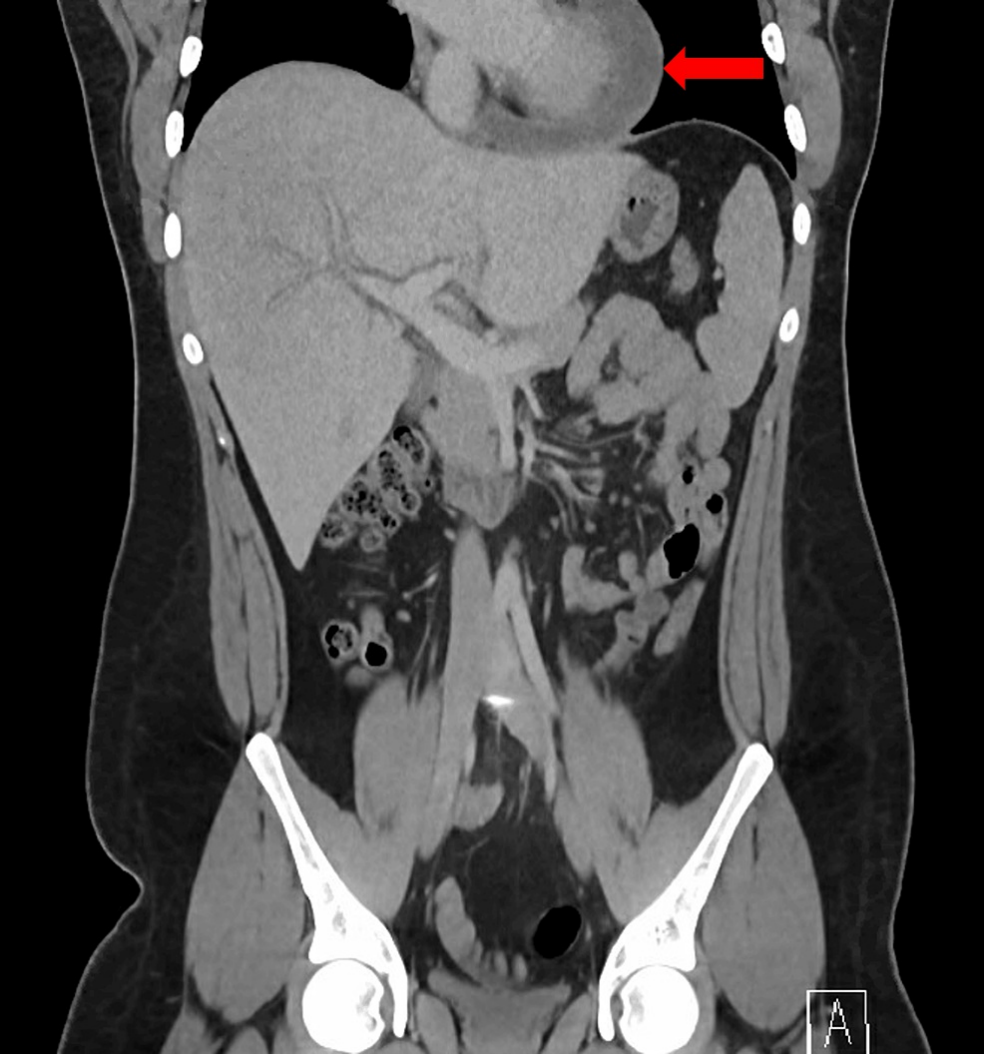
CT scan showing hepatomegaly, splenomegaly, and pericardial effusion (red arrow).

**Figure 2:**
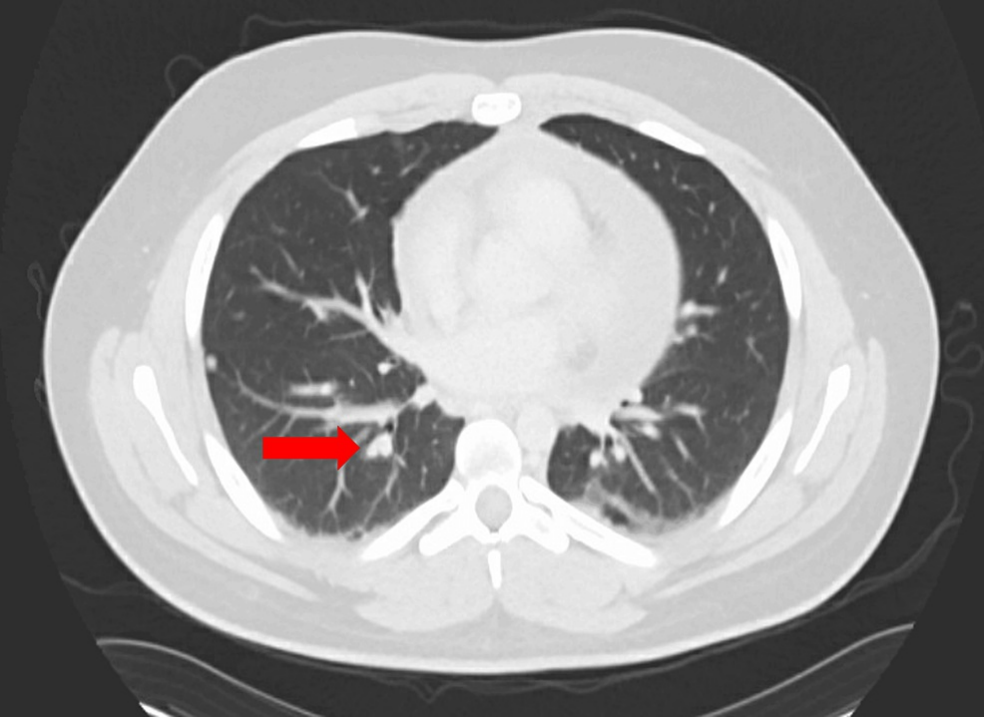
CT scan showing a small right pulmonary nodule (red arrow).

Unfortunately, the pain persisted and worsened over the next two weeks, and he developed fevers (reported 38-39°C), generalized aches, and increasing fatigue. He again presented to the emergency department with these symptoms. On presentation, he was tachycardic (heart rate: 132 beats/minute), normotensive (blood pressure: 132/60 mmHg), and with a temperature of 37.4°C which shortly increased to 39.3°C. Physical examination noted tachycardia and diffuse abdominal tenderness. EKG showed sinus tachycardia and a nonspecific T-wave abnormality. Basic laboratory studies showed mild hyponatremia (134 mmol/L), hyperbilirubinemia (2.6 mg/dL), mild leukocytosis (13.5K/µL), and new anemia (hemoglobin 11.4g/dL from 14.4 g/dL three weeks prior). Significant inflammation was noted (C-reactive protein: 305 mg/L, erythrocyte sedimentation rate: 104 mm/hour). Transaminases were normal. Abdominal imaging revealed hepatomegaly, mild splenomegaly, pericardial effusion with some enhancement (Figure 1), and a 4-5 mm noncalcified pulmonary nodule in the lateral right upper lobe (Figure 2).

Echocardiography showed a normal ejection fraction, no significant valvular dysfunction, and a small circumferential pericardial effusion with no evidence of tamponade. He was started on ceftriaxone for possible bacterial infection and received several liters of parenteral fluids. Following fluid resuscitation, his hemoglobin was 9.8 g/dL. The human immunodeficiency virus test was negative.

He was diagnosed with pericarditis and started on scheduled indomethacin and colchicine. Following the initiation of the medication, he had no further fevers but continued to have diffuse pain and malaise. At this point, further history was obtained. Shortly before his initial symptoms began three weeks prior, he was helping his parents clean out a bird coop that housed chickens and turkeys. Based on his negative bacterial cultures and lack of overall improvement, his ceftriaxone was discontinued. With a new concern for fungal infection, he was given one dose of liposomal amphotericin B 10 mg/kg followed by itraconazole. The serum cryptococcal antigen was negative. Two days after the dose of amphotericin, he noted some symptomatic improvement and return of appetite. He was discharged from the hospital on indomethacin and colchicine for pericarditis and itraconazole as empiric antifungal therapy.

He continued slow improvement after dismissal, although he did develop loose stools which improved with cessation of colchicine. Repeat echocardiography one week after hospital discharge showed resolution of the pericardial effusion. At this point, his fungal testing returned, and *Histoplasma *immunodiffusion testing showed the presence of an M band only. *Histoplasma *complement fixation titer was positive for the yeast phase at 1:128 (normal: <1:8) and mycelial phase at <1:8. Urinary *Histoplasma* antigen was negative. He was continued on itraconazole. As he was completely asymptomatic at follow-up seven weeks after hospital discharge, antifungal therapy was discontinued.

## Discussion

While *Histoplasma capsulatum* infection may frequently present as acute pneumonia or pulmonary granulomas, multiple different clinical presentations are possible. Once the microconidia have been inhaled, they are ingested by macrophages and neutrophils in an attempt for the body’s immune system to prevent widespread infection [2]. While macrophages are generally the primary defense mechanism against histoplasmosis, both natural killer cells and T cells play a role in limiting the extent of infection [3]. However, *Histoplasma capsulatum* has multiple virulence factors that allow it to survive the host defense mechanisms, beginning with avoiding intracellular destruction and replicating inside macrophages [4]. In immunocompetent hosts, cytokines (typically interferon-gamma, tumor necrosis factor-alpha, and interleukin-17) are generally able to contain the fungal elements in their more common granulomatous and indolent state. Immunocompromised individuals are at a higher risk for reactivation of this granuloma at any time [2]. Those with intact immunity are not safe from extensive infection, but rather it typically takes a higher inoculum of fungus to cause a more severe infection [4]. This makes it particularly important to differentiate the etiology of a granuloma before treatment, given that corticosteroids erroneously given for some diseases (i.e., sarcoidosis) can exacerbate an underlying previously subclinical histoplasmosis infection [5].

Pericarditis caused by *Histoplasma capsulatum* has been reported, both caused by local inflammation of mediastinal lymph nodes as well as direct invasion of the infection into the pericardium [6]. One case reported a formerly healthy 19-year-old female who developed pericarditis with effusion with concomitant coarctation of the aorta. Her pericardial biopsy showed a granuloma caused by the organism itself [7]. Another similar case of an adult male with acute pericarditis with a diffusely thickened pericardium was diagnosed when coronary artery bypass grafting revealed necrotizing granuloma formation and positive fungal biopsy [5]. However, other cases of pericarditis have been attributed to *Histoplasma *without such obvious test results. In one such case, a young 19-year-old healthy male developed pericarditis, refractory to initial management with subsequent tamponade, and eventually found to have a lung nodule with budding yeast consistent with *Histoplasma *requiring antifungal therapy with which he finally showed improvement. In this case, the patient’s pericardial fluid showed no growth of fungal organisms, and pericardial biopsy revealed chronic inflammation and fibrosis without granuloma formation as in some other cases of pericarditis attributed to *Histoplasma *[8].

Reports of *Histoplasma *pericarditis have yielded both obvious results for *Histoplasma capsulatum* being the culprit as well as only general inflammation. The likely pathophysiology can be attributed to robust inflammation induced by the organism in general, given that several mechanisms of the immune system are triggered upon infection. It is thought that there is some degree of hypersensitivity within the local mediastinal lymph nodes to the *Histoplasma *antigens [5]. Regional inflammation that often occurs during acute infection can cause pericardial irritation and resultant inflammation [2]. In an immunocompetent host, without direct inoculation of the organism into the pericardium, the yield of an organism via biopsy or fluid studies is likely to be low. Data gathered during large urban outbreaks of histoplasmosis in Indianapolis suggest that pericarditis may occur in over 6% of acute symptomatic histoplasmosis cases [9].

As the patient presented in this case did have environmental exposure that placed him at a higher risk for fungal infection, this is an example of a patient in whom it is reasonable to consider histoplasmosis as an underlying cause for pericarditis. While basic imaging did not suggest overt inflammation or invasion from mediastinal lymphadenopathy, there were no pericardial fluid studies or biopsies performed to definitively rule in or out granulomatous or fungal involvement. The patient’s immune response (given elevated inflammatory markers) suggests a marked systemic inflammatory response, while negative urine antigen testing made true disseminated histoplasmosis less likely. In addition, the high *Histoplasma *yeast complement fixation titer also supports acute infection as being related to his pericarditis. It is noteworthy to point out that the patient had a negative urinary antigen despite having positive complement fixation titers. Antigenemia or antigenuria is diagnostic for infection as it shows the clear presence of the organism. However, it is possible to have limited or localized infection without antigenemia or antigenuria, diagnosed by evidence of host response to the organism, as measured by serologic testing.

In general, pericarditis is often benign without an etiology found in most cases [10]. While often attributed to viral infections when cultures are negative, other non-infectious etiologies can cause pericarditis such as malignancy, rheumatologic disorders, and medications. When the answer remains unclear or patients have other symptoms, it is important to remember *Histoplasma capsulatum* as a possible causative agent, particularly in endemic areas. As systemic corticosteroid use may increase the risk of disseminated histoplasmosis, this diagnosis should be considered in patients with pericarditis who are to receive this therapy due to poor response, intolerance, or contraindication to nonsteroidal anti-inflammatory drug therapy.

## Conclusions

While healthy hosts frequently do not suffer from severe infection from histoplasmosis, complications such as disseminated infection or nonpulmonary manifestations can be seen. Pericarditis can be a symptom of an inflammatory reaction caused by histoplasmosis. Generalized inflammation may occur as a result of the host response to the fungal infection. It is important to keep histoplasmosis on the differential when patient symptoms are unclear to prevent missing a diagnosis and allow for the initiation of earlier treatment.
